# Quantifying Glioblastoma Drug Response Dynamics Incorporating Treatment Sensitivity and Blood Brain Barrier Penetrance From Experimental Data

**DOI:** 10.3389/fphys.2020.00830

**Published:** 2020-08-21

**Authors:** Susan Christine Massey, Javier C. Urcuyo, Bianca Maria Marin, Jann N. Sarkaria, Kristin R. Swanson

**Affiliations:** ^1^Precision Neurotherapeutics Innovation Program, Mayo Clinic, Phoenix, AZ, United States; ^2^Department of Radiation Oncology, Mayo Clinic, Rochester, MN, United States; ^3^Department of Neurological Surgery, Mayo Clinic, Phoenix, AZ, United States; ^4^School of Mathematical and Statistical Sciences, Arizona State University, Tempe, AZ, United States

**Keywords:** glioblastoma, blood–brain barrier, drug sensitivity, epidermal growth factor receptor (EGFR), parameter estimation

## Abstract

Many drugs investigated for the treatment of glioblastoma (GBM) have had disappointing clinical trial results. Efficacy of these agents is dependent on adequate delivery to sensitive tumor cell populations, which is limited by the blood-brain barrier (BBB). Additionally, tumor heterogeneity can lead to subpopulations of cells with different sensitivities to anti-cancer drugs, further impacting therapeutic efficacy. Thus, it may be important to evaluate the extent to which BBB limitations and heterogeneous sensitivity each contribute to a drug's failure. To address this challenge, we developed a minimal mathematical model to characterize these elements of overall drug response, informed by time-series bioluminescence imaging data from a treated patient-derived xenograft (PDX) experimental model. By fitting this mathematical model to a preliminary dataset in a series of nonlinear regression steps, we estimated parameter values for individual PDX subjects that correspond to the dynamics seen in experimental data. Using these estimates as a guide for parameter ranges, we ran model simulations and performed a parameter sensitivity analysis using Latin hypercube sampling and partial rank correlation coefficients. Results from this analysis combined with simulations suggest that BBB permeability may play a slightly greater role in therapeutic efficacy than relative drug sensitivity. Additionally, we discuss recommendations for future experiments based on insights gained from this model. Further research in this area will be vital for improving the development of effective new therapies for glioblastoma patients.

## 1. Introduction

Glioblastoma (GBM) is an aggressive primary brain cancer that is notoriously difficult to treat due to its diffuse infiltration into surrounding normal-appearing brain (Giese et al., 2003). These diffusely invading GBM cells cannot be completely resected surgically (Baldock et al., 2014), and are difficult to target with radiation therapy while sparing normal brain (Corwin et al., 2013). As a result, clinicians rely on chemotherapy to treat the full extent of the tumor. However, chemotherapeutic efficacy can be limited in two main ways: there may be insufficient delivery across the blood–brain barrier (BBB), and the tumor may not be uniformly sensitive to the agent.

The BBB acts to keep pathogens and many toxins out of the sensitive brain tissue. Angiogenesis in dense tumor regions induces disruption of the BBB, potentially allowing chemotherapeutic drugs to “leak” into these tumor regions. Current dogma in neuro-oncology holds this as being largely sufficient to treat the tumor, but GBM cells invade beyond these regions into tissue where the BBB remains rather intact (Van Tellingen et al., 2015). Further, tissue interstitial pressure and drug properties such as lipophilicity and polarity may influence the delivery of drugs across angiogenesis-induced BBB “leaks” (Ningaraj, 2006). Due to these factors, it remains unclear whether the delivery of BBB-impermeable antineoplastic agents reaches adequate concentrations throughout the tissue to provide the anticipated therapeutic effect.

Drug insensitivity (which includes, but is not limited to resistance) of tumor subpopulations is also a key suspect behind unsuccessful molecularly-targeted therapy results (Wen and Kesari, 2008; Ene and Holland, 2015). GBMs frequently present with gene mutations and/or amplification for a number of targets, such as epidermal growth factor receptor (EGFR). However, due to the spatial heterogeneity of GBM, these targets may have been identified for a subpopulation that is predominant in the dense tumor core, but less common in the invading portions of the tumor. There may also be activated compensatory signaling pathways in some cells or tumor regions that confer reduced responsiveness to the drug. Thus, while therapies already exist for molecular targets identified in GBM (Nagane et al., 1996; Brennan et al., 2013; Eskilsson et al., 2017; Reardon et al., 2017; van den Bent et al., 2017), a significant proportion of tumor may be less sensitive to these drugs, potentially explaining why they have failed in clinical trials (de Groot et al., 2008; De Witt Hamer, 2010; Reardon et al., 2010; AbbVie, 2019). However, it has been difficult to separate the possible insensitivity related causes of drug failure from that of inadequate delivery across the BBB and distribution throughout the tumor, since the majority of these drugs were not developed specifically for brain.

In order to explore both the contributions of inadequate delivery of therapy across the BBB and drug insensitivity, we developed a minimal mathematical model based on experimental data from preclinical subjects treated with an EGFR-targeted antibody drug conjugate (ADC). First, we describe model development based on this data, which consists of two tumor subpopulations with high vs. low sensitivity to the ADC therapy, and steps to estimate parameter regimes via data-fitting. Next, we explore the global model parameter sensitivity to understand how these parameters impact model outcomes. Finally, we run model simulations for the data-derived parameter regimes to assess the relative contributions of drug distribution and sensitivity, and discuss how it might be useful in assessing results from future experiments comparing different tumor models or different drugs. Overall, our model suggests that the degree of drug exposure may be more impactful than the relative sensitivity to therapy between the tumor subpopulations. Thus, in order to improve treatment outcomes, it is critical to determine predictors of drug distribution in individual patients' tumors and surrounding brain tissue to ensure invading tumor cells are adequately exposed to the therapy.

## 2. Methods

Our ordinary differential equation (ODE) model of tumor growth and treatment response accounts for both variable treatment exposure and differential sensitivity to treatment by different tumor subpopulations. Development of this model was informed by experimental observations, which were also used to determine relevant parameter regimes for running simulations.

### 2.1. Experimental Data

**Figure 1:**
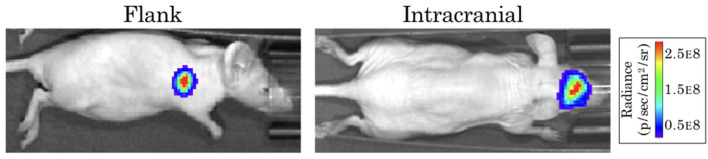
Example bioluminescence images for patient-derived xenografts. Colors represent the BLI radiance (in photons/second/cm^2^/steradian), which is related to the BLI flux (measured in photons per second) for the total area.

The form of our model was based on experimental data from testing an EGFR-targeted antibody drug conjugate (ADC) in a patient-derived xenograft (PDX) model of GBM (Marin et al., 2018). These experiments were performed in full accordance with the guidelines of the Mayo Clinic Institutional Animal Care and Use Committee. The GBM12 PDX line used in this model is derived from a primary GBM in a male patient, and is EGFR amplified, MGMT methylated, and IDH1 and IDH2 wildtype. Full detail regarding this PDX line is available from the Mayo Clinic Brain Tumor Patient-Derived Xenograft National Resource (https://www.mayo.edu/research/labs/translational-neuro-oncology/mayo-clinic-brain-tumor-patient-derived-xenograft-national-resource/), where the line was developed and is maintained. These cells were implanted intracranially into 10 female athymic nude mice, and into the flank of 10 additional mice. After tumors were established, a subset of the surviving mice in each group was treated with either 10 mg/kg of a sham control antibody (Ab-095; four mice in each), or with 5 mg/kg of ABT-414 (an ADC also known as depatuxizumab mafodotin; five mice in each), administered via tail vein injection every 7 days. Tumor growth was monitored via bioluminescent imaging (BLI, Figure 1). Since BLI flux is *linearly* correlated with tumor cell number (Hartung et al., 2014), this provided us with a close approximation of tumor cell populations across time. Importantly, the data from PDX tumors grown in the flank and brain allowed us to compare treatment effect in tumors with and without BBB impediments to drug distribution.

### 2.2. Treatment Exposure and Sensitivity Model

Our model consists of three coupled ordinary differential equations describing the dynamics of both cell populations (*H*, *L*) and the ADC (*A*):

(1a) $$\begin{array}{l} \frac{dH}{dt}=\underset{\text{proliferation}}{\underset{︸}{\rho H}}-\underset{\text{drug-induced apoptosis}}{\underset{︸}{\gamma \mu_{H}AH}} \end{array}$$

(1b) $$\begin{array}{l} \frac{dL}{dt}=\underset{\text{proliferation}}{\underset{︸}{\rho L}}-\underset{\text{drug-induced apoptosis}}{\underset{︸}{\gamma \mu_{L}AL}} \end{array}$$

(1c) $$\begin{array}{l} \frac{dA}{dt}=\underset{\text{drug dose given at time}t}{\underset{︸}{\overset{N}{\underset{n=1}{\sum }}A_{\text{dose}}\left(n\right)\delta \left(t-7n\right)}}-\underset{\text{drug decay }}{\underset{︸}{\lambda A}} \end{array}$$

where parameters and their definitions are outlined in Table 1, and their derivations can be found in section 2.3.

**Table 1:** Model parameter definitions and values.

**Symbol**	**Definition**	**Value range**	**Units**
ρ	cellular proliferation rate	0.2–0.5	day^−1^
μ_*H*_	ADC-mediated high sensitivity cell kill rate	1–10	mg^−1^day^−1^
μ_*L*_	ADC-mediated low sensitivity cell kill rate	*zμ*_*H*_	mg^−1^day^−1^
*q*	proportion of implanted cells with low sensitivity	0–1	unitless
*z*	relative sensitivity (μ_*L*_/μ_*H*_)	0–1	unitless
λ	rate of ADC decay	ln(2)/7	day^−1^
γ	proportion of tumor exposed	0–1	unitless
*A*_dose_	ADC given in a single dose	0.1	mg

*Parameter value ranges were estimated through fitting the model to experimental data or parameters were confined to a value range by their theoretical meaning, except in the case of ADC parameters, as described in section 2.3*.

**Figure 2:**
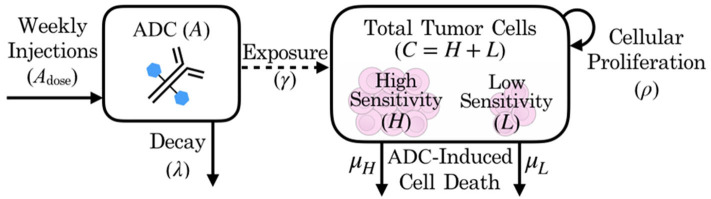
Schematic of patient-derived xenograft response to an antibody drug conjugate, including key variables and parameters of the mathematical model.

In the absence of the ADC, both highly sensitive (*H*) and less-sensitive (*L*) tumor populations grow exponentially, at proliferation rate ρ. However, the two populations differ in sensitivity to the ADC, *A*, which is captured by the drug-induced apoptosis rates μ_*H*_ and μ_*L*_ (for populations with high and low sensitivity, respectively). The terms for tumor cell death due to ADC are further modified by factor γ, which represents the proportion of cells exposed to ADC. We assume that the ADC is readily distributed to flank PDXs such that tumor cell exposure is high (γ = 1), but that the BBB limits this distribution for intracranial PDXs (0 ≤ γ ≤ 1). In order to capture the ADC dynamics, we let *A*_dose_(*n*) represent the *n*th dose, with doses administered every seven days, as noted by the dirac delta function δ(*t* − 7*n*). The ADC then decays at rate λ. These dynamics are schematized in Figure 2.

The model as described incorporates several assumptions that are useful to note explicitly. (1) While in theory there may be many groups of cells with varying levels of therapeutic sensitivity (some of which may even encapsulate resistant subpopulations), the model divides these into two main groups: those of relatively high therapeutic sensitivity and those with lower sensitivity to the therapy. (2) Any drug effect on proliferation rate is captured by the cell death rate parameter, as these effects are indistinguishable with the available data. (3) The effects of the tissue environment in the flank vs. the brain on tumor *growth* are encapsulated in environment-specific cellular proliferation rate parameter values (ρ_flank_ and ρ_IC_, as described in section 2.3). (4) Relatedly, the cell death rate due to therapy for the sensitive tumor subpopulation, μ_*H*_, is assumed to be the same intracranially as in the flank setting, only modified by drug exposure after crossing the BBB in intracranial tumors. That is, the model assumes that the only environmental effect on *treatment* is its distribution. (5) ADC and tumor subpopulations are well-mixed. In reality there is likely spatial variation in both flank and intracranial sites due to the tumor microenvironment and different blood vessel densities in particular tumor regions, but in the absence of spatially–resolved data, we assume well-mixedness and use an ODE model.

This model can be solved analytically, as shown in Appendix. For simplification, at any given time *t*, *C*(*t*) represents the total number of cells, calculated by the sum of high sensitivity *H*(*t*) and low sensitivity *L*(*t*) cells. This total cell number was used in section 2.3 for comparing with bioluminescence imaging data, which shows the total tumor cell population. The initial proportion of total implanted cells with low sensitivity is denoted by *q* = *L*_0_/*C*_0_. Similarly, the extent to which these cells *L* are less sensitive to the agent than the highly sensitive cells *H* is denoted by the relative sensitivity ratio *z* = μ_*L*_/μ_*H*_, which is bounded between 0 and 1 to ensure that μ_*L*_ is a fraction of μ_*H*_ in the regression-based parameterization in section 2.3. With these notational changes, we can then write the analytical solution (derived in Appendix) as

(2) $$\begin{array}{l} C\left(t\right)=C_{0}e^{\rho t}\left(qe^{-\gamma \mu_{H}\int A\left(t\right)dt}+\left(1-q\right)e^{-\gamma z\mu_{H}\int A\left(t\right)dt}\right), \end{array}$$

where

(3) $$\begin{array}{l} \int A\left(t\right)dt=\overset{N}{\underset{n=1}{\sum }}2^{n}A_{\text{dose}}\left(n\right)\frac{\left(e^{7n\lambda }-e^{\lambda t}\right)\theta \left(t-7n\right)}{\lambda }. \end{array}$$

Using this solution (2), the model can be parameterized through comparison of simulations to time-series BLI data.

### 2.3. Data-Based Parameter Estimation

Most model parameters were unknown, with the exception of ADC-specific parameters: the timing of dose administration and dose amounts [*A*_dose_(*n*)], as well as the half-life of the drug, which allowed us to solve for the drug decay rate (λ). Dose amounts were adjusted for the weight of each animal (5 mg/kg), so we applied the average initial animal weight of 20 g to obtain the constant ADC dose, *A*_dose_ = 0.1 mg used in simulations. All of the remaining model parameters were determined through several iterations of fitting the model via least squares regression to preliminary BLI data from an experiment. The various arms of the experiment included untreated and treated groups of subjects, as well as flank and intracranial tumor sites to separate out BBB influences. By fitting the model to these various subgroups, we were able to identify and estimate each of the parameters, as described below.

**Figure 3:**
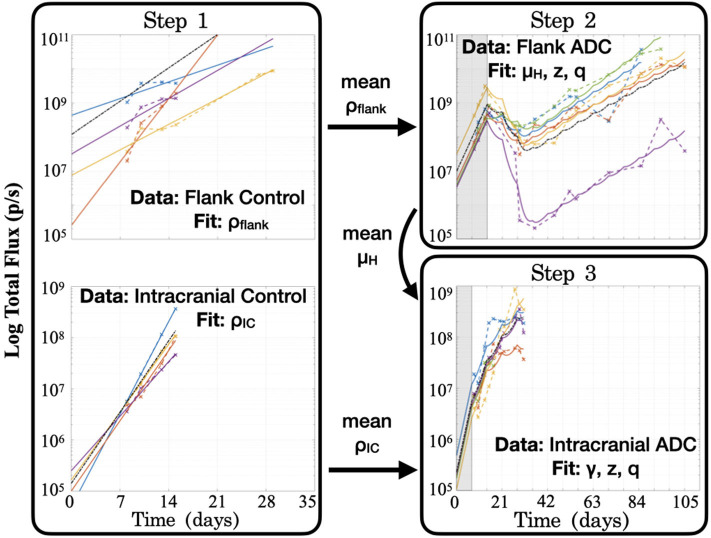
Summary of the series of steps used to estimate model parameter values through fitting the model to different experimental data sets. **Step 1** consists of two separate fittings for the two different tumor locations, and the mean ρ values are passed for use in subsequent steps in the respective tumor sites, while the mean μ_*H*_ value from the flank found in **Step 2** is passed for use in **Step 3**. Shaded regions for Steps 2 and 3 indicate time prior to initiation of treatment with the ADC. Black dash–dot lines in each plot are simulations using the averaged fitted parameter values across all subjects within the group. It is worth noting that at each step, no more than three parameters are fitted to the data, in order to ensure identifiability and prevent overfitting.

**Step 1: Fit to untreated data to estimate growth rate**, ρ. When fitting the model to untreated data, since the ADC is not injected (*A* = 0), the model's treatment components zero out and only an exponential growth function remains: $C\left(t\right)=C_{0}e^{\rho t}$. Fitting this model function to untreated data via least squares regression with the lsqcurvefit function in Matlab**®** (MATLAB Release 2018b, The MathWorks, Inc., Natick, Massachusetts, United States), we were able to obtain estimates of the tumor proliferation rate, ρ, and the number of viable implanted PDX cells, *C*_0_ (Figure 3). (While a consistent number of cells are initially implanted for each subject, *C*_0_ is in fact unique for each, as a variable number of cells die off, possibly due to an inability to establish themselves in the proper microenvironment for growth.) This yielded subject-specific values for ρ and *C*_0_ (which were bound on the intervals [0, inf) and [10^2^, 10^10^], respectively), and the mean ρ was recorded as the net proliferation rate for the cells of the particular PDX line used in the experiments grown in either the flank (ρ_flank_) or intracranial (ρ_IC_) setting. As noted in the model assumptions, these site–specific ρ parameter values from untreated tumors are used to help to account for microenivironmental effects on PDX growth in the two different locations that are independent of the BBB.

**Step 2: Fit to treated flank data to estimate** μ_*H*_, *z***, and** *q*. Using the estimated net proliferation rate ρ_flank_ from the previous step, we proceed to fit the treated data in the flank. We assume the estimate of ρ_flank_ remains the same in the treated case as untreated, since the microenvironment remains similar and any differences should be encapsulated in the treatment effect term. Additionally, since the tumor was injected in the flank, there is no BBB effect to limit the proportion of tumor exposed to the ADC, such that the exposure parameter γ = 1. The initial condition *C*_0_ is fit using the initial untreated time point and the passed mean ρ_flank_ value. Pairing these with other known parameters (see Table 1), the only remaining three unknown parameters to be fit to the data are the cell death rates due to drug, μ_*H*_ and μ_*L*_, for the two cell populations and the proportion of implanted cells that have low sensitivity, *q*. Using the definition *z* = μ_*L*_/μ_*H*_ and the analytical solution of the model (2), we can then apply a nonlinear least squares regression (again using lsqcurvefit) to fit subject-specific parameters for parameters μ_*H*_, *z*, and *q* (bound on the intervals [1, 10], [0, 1] and [10^−10^, 10^−2^], respectively).

**Step 3: Fit to treated intracranial data to estimate** γ, *z***, and** *q*. Proceeding to fit the data from treated intracranial tumors, we apply the same approach to estimate parameters as in the flank, this time assuming that the estimate of ρ_IC_ from the untreated setting remains the same for the treated intracranial tumors due to a similar microenvironment. Again we determine the initial condition *C*_0_ by fitting the untreated model with the passed mean ρ_IC_ value to the initial untreated time point. Because we assume that the cell death rate due to ADC for the highly sensitive tumor subpopulation (μ_*H*_) is the same intracranially as in the flank setting, we pass the average μ_*H*_ value determined in Step 2 and estimate parameter γ, the fraction of tumor exposed to therapy, in addition to parameters *z* and *q*.

At the conclusion of these steps (summarized in Figure 3), all unknown model parameters had net and individual estimates. Note that no more than three parameters were fitted with any experimentally-derived data set, in order to reduce the potential for overfitting. To examine this further, initial parameter guesses for lsqcurvefit were selected from within the value ranges listed in Table 1, and we had mostly consistent convergence using low, middle, and high values (see Supplementary Material). Additionally, as we show in the Figure 3 plots with dash–dot lines, the simulations that result when using the averages of the fitted parameter values across all the subjects capture the dynamics of the data well. Thus, the averaged ρ_flank_, ρ_IC_, and μ_*H*_ values that we pass for later fitting steps correspond well with the group as a whole. Using these values then allowed us to run simulations in a reasonable range of parameter values, as well as to perform a model sensitivity analysis to understand how variability in these values affect model outcomes.

## 3. Results

### 3.1. Parameter Sensitivity Analysis

**Figure 4:**
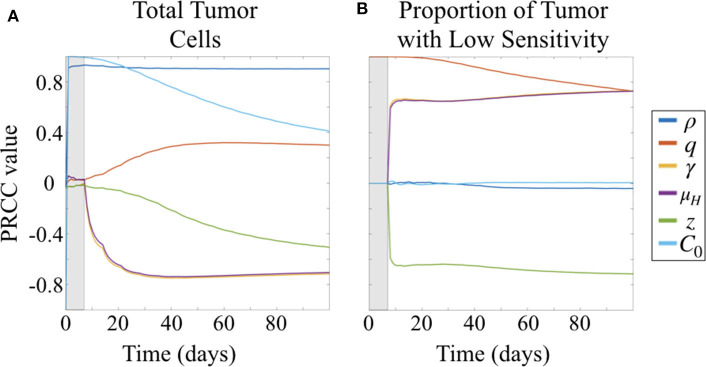
Partial rank correlation coefficients (PRCC) of parameters with respect to **(A)** tumor cells and **(B)** fraction of cells with low sensitivity (*L*/(*H* + *L*) = *L*/*C*), visualized across simulation time.

Due to the uncertainty and variability in our parameter estimates, it was important to better characterize the effects of parameters on model results. To do this, we conducted a parameter sensitivity analysis via Latin hypercube sampling (LHS) and partial-rank correlation coefficients (PRCC) (McKay et al., 1979; Iman and Helton, 1988; Blower and Dowlatabadi, 1994). To perform the LHS analysis, we first drew 1,000 equiprobable samples for each unknown parameter, including the initial condition *C*_0_, from a statistical distribution of values. These distributions were informed by our fits of the preliminary data when available; in the case of the unitless parameters, we assumed a uniform distribution on the interval [0, 1]. These samples were then randomly paired in a Latin hypercube scheme to run a series of 1,000 Monte Carlo simulations. Using these simulation results, we then computed PRCCs between each parameter and two different model outcomes across all time points: the total number of tumor cells and the fraction of tumor that has low sensitivity (Figure 4). PRCC are computed using partial correlation applied to value ranks, as opposed to the actual values of the parameters and model outcome. Partial correlation helps control for effects due to other covariates, and ranks are used to evaluate the associative relationship between high and low values of parameters and the model outcomes, rather than the values themselves. Thus, the PRCC values at a given time point indicate how closely a high model outcome value relates to a high or low parameter value given at that time in the simulation. A PRCC close to 1 indicates a strong association between high parameter value and high model outcome value, and a PRCC close to −1 indicates a strong association between a low parameter value and a high model outcome value. For PRCC values between −0.5 and 0.5, the association is considered to be weak. Further details for about this method and the code files used are available on GitHub: https://github.com/scmassey/model-sensitivity-analysis.

#### 3.1.1. Total Tumor Population Depends Most Strongly on Proliferation Rate, Followed by Treatment Response Parameters

At early time points, particularly before the initiation of therapy, the tumor population is strongly positively correlated with both the initial number of cells implanted, *C*_0_ and proliferation rate ρ (Figure 4A). By 30 days, or after approximately three doses of therapy, the population remains strongly positively correlated with ρ but the effect of *C*_0_ begins to wane. At the same time, drug sensitivity of the *H* cell population, μ_*H*_, and exposure to drug, γ are strongly negatively correlated with total tumor cells. Relative sensitivity *z*, which determines the fraction of drug sensitivity in the *L* cell population, is also negatively correlated with total tumor, but less strongly, and only approaches a PRCC value of −0.5 after 100 days. This suggests that relative sensitivity *z* is less impactful than either the treatment response rate μ_*H*_ for the subpopulation with high ADC sensitivity or the degree of tumor exposure to ADC, γ.

Parameters γ and μ_*H*_ track together in the sensitivity analysis (overlapping lines in Figure 4). This is expected given our substitution μ_*L*_ = *zμ*_*H*_, which results in the coefficient −γ*zμ*_*H*_ in the term describing drug induced apoptosis for the equation describing the *L* population (Equation 1b), mirroring that for the *H* population (Equation 1a), −γμ_*H*_. Thus, sensitivity analysis is unable to compare the differential impacts of these two parameters, and highlights a potential parameter identifiability issue for our model. Since we had preliminary data in both the treated flank as well as the treated intracranial PDX settings, we were able to obtain parameter estimates for these by keeping γ = 1 in the flank setting, and assuming that μ_*H*_ is the same intracranially as in flank.

#### 3.1.2. Less Sensitive Fraction of Tumor Driven by Initial Proportion of These Cells, Followed by Treatment Response Parameters

Prior to the initiation of therapy, only parameter *q*, the fraction of initially implanted cells that have low sensitivity, is correlated with the proportion of total tumor that has low sensitivity (Figure 4B). Once treatment is initialized, *q* remains highly positively correlated, and this correlation decreases slightly over time during the course of treatment.

Three other parameters show correlation with the fraction of tumor that has lower sensitivity following initiation of therapy, all of which involve drug response. Parameters γ and μ_*H*_, representing the degree of tumor exposure to ADC and the ADC-induced cell kill rate of cells with high sensitivity, respectively, are both positively correlated and track together, while parameter *z*, representing the relative treatment sensitivity between the cells with higher and lower sensitivity, is negatively correlated with the fraction of tumor that has low sensitivity. Further, the PRCC values do not vary over the time of the simulation after treatment is initiated and sustained. These correlations are consistent with expectations from the behavior of the system described by the model.

### 3.2. Simulation Results

To more fully explore the effect of parameters on model predicted outcomes, we ran simulations for varied values of the parameters relating to treatment response: γ, μ_*H*_, and *z* (degree of ADC exposure, the ADC response rate in cells with high sensitivity, and the relative sensitivity between the two tumor subpopulations, respectively). Codes used to run simulations and plot the results may be found on GitHub: https://github.com/scmassey/treatment-exposure-sensitivity-model.

#### 3.2.1. Treatment Exposure Impacts Tumor Burden More Than Relative Sensitivity

Comparing simulation results across a range of values for parameters γ and *z* while holding μ_*H*_ fixed, we see that γ plays a larger influence on total tumor cells than does *z*. That is, looking across rows of drug exposure values γ, we see that for relative sensitivity *z* > 0.6, there is no variation in total tumor burden. For lower levels of drug exposure, this is even more pronounced, as tumor burden is quite high regardless of the relative sensitivity. This is consistent with the parameter sensitivity results of section 3.1.1, but shows the impact of this dynamic in greater detail.

#### 3.2.2. Different Subpopulation Proportions Can Yield Same Total Tumor Burden

**Figure 5:**
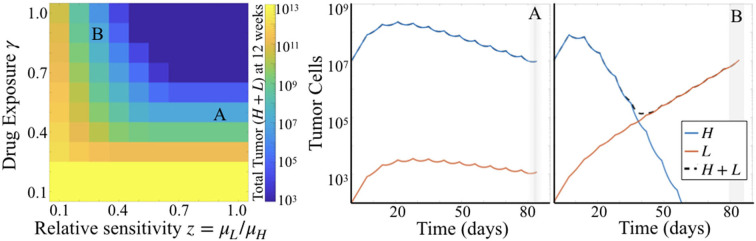
Simulation results for varied γ and *z*. **(Left)** Heatmap shows total tumor cells at 84 days (12 weeks) post tumor initiation, across 10 values each of γ and *z*, for fixed μ_*H*_ = 5. **(Right)** Two particular simulations corresponding to the labels (A,B) in the heatmap.

Simulations also highlighted that there can be distinct differences in the dynamics of the two subpopulations of cells underlying predicted tumor burden (Figure 5). Looking at long time scales—in this case at 12 weeks or 84 days, the average survival time of the treated subjects—we observe the effect of an extended time of treatment in the simulations. Comparing two simulations with the same predicted tumor burden, we see that one simulation retains a large proportion of cells with high sensitivity (Figure 5A), while another is made up almost entirely of cells with low treatment sensitivity (Figure 5B). Thus, while the overall tumor may look similar at many points along its trajectory if sampled sparsely, one is about to be “uncontrolled” at later time points, while the other will stay relatively stable.

## 4. Discussion

Failure of targeted therapies in glioblastoma can be attributed to many different causes, many of which are driven by various aspects of tumor heterogeneity. These include the potential mismatch of treatment to target beyond the center of the tumor and/or inadequate delivery of therapy to these cells invading outlying brain parenchyma. Often these have been investigated separately, focusing either on sensitivity (through optimizing targets or overcoming resistance) (Cloughesy et al., 2014), or engineering approaches for enhancing drug delivery (Liu et al., 2010; Van Tellingen et al., 2015). Infrequently, elements of both are combined (Stein et al., 2018), but even in those cases the relative impact of these upon treatment outcomes has not been compared. Thus, we created our Treatment Exposure and Sensitivity model describing tumor growth and treatment response incorporating both exposure and differential sensitivity to therapy, based on experimental data, to investigate the relationship between them. Through parameter sensitivity analysis and simulation, we found that both can contribute in similar ways, but exposure may have a greater impact overall. In particular, for simulated tumors that were given the same treatment responsiveness for the highly sensitive population, therapeutic exposure impacts tumor burden more than the relative sensitivity between the two tumor populations. Sensitivity analysis also revealed that parameter ρ is the strongest positive influence on total cell population, as expected, and distinguished between the effect of reduced therapeutic sensitivity (*z*) and exposure to therapy (γ) in reducing the total tumor population. Not only is there a difference in the magnitude of correlation between parameters γ and *z* with total tumor burden, there is also a difference in the temporal dynamics of the change in these correlations over time. The correlation coefficients between parameters and the fraction of the tumor with lower treatment sensitivity, however, is quite stable over time.

### 4.1. Limitations

Our model is relatively minimal by design, as the amount of available data constrains the number of model parameters we can fit. Thus, this model does not compare potential sources of differential treatment sensitivity (such as various mechanisms of resistance). Although tumor heterogeneity may actually provide for many populations with varying levels of sensitivity to therapy, as described in section 2.2 we assumed that these cluster toward more or less sensitive, reducing them to two. Larger data sets generated by similar studies in the future may support including more populations and additional mechanistic differences or interactions between them. Related to this, we were also limited in distinguishing BBB impacts from other microenvironmental effects when comparing data from tumors grown in the flank vs. the intracranial setting beyond the proliferation rate, ρ. For example, microenvironmental effects, such as vascular density, likely create regions of differential drug exposure (as well as regions of different tumor subpopulations). Experiments generating larger data sets and greater spatial detail would facilitate adding these features in future modeling efforts. Finally, while our present analysis of the model enables us to understand the overall dynamics between the parameters, we are not able to conclude anything with respect to efficacy of this particular drug among patients due to the use of a single PDX line. As we discuss below, however, by using the parameter fitting method presented to estimate drug sensitivity and exposure across multiple PDX lines in future studies, it may be possible to gain insight in these factors across patients with this heterogeneous disease.

### 4.2. Model Recommendations for Future Experiments

Parameter sensitivity analysis of the model highlighted the necessity of having the flank and intracranial treatment groups for practical identifiability in obtaining estimates for therapeutic sensitivity (μ_*H*_) and exposure (γ). This “tradeoff” between γ and μ_*H*_ was also observed in simulations and is expected given our model formulation. However, the emergence of this dynamic in the creation and parameter estimation of our model underscores that this relationship should be carefully considered in the design of experimental studies for new glioma therapies. Sensitivity analysis revealed that correlation coefficients between parameters and total tumor burden change most dramatically at earlier time points, and after approximately 50 days, change relatively little. This suggests that experiments conducted to examine the relationship between exposure and sensitivity to therapy should focus on collecting time course data more densely for the first 7 weeks as compared to longer times. As shown by simulations, there can be several parameterizations that fit tumor burden data at any single time which correspond to different proportions of cells with high and low sensitivity to treatment. Thus, time series data is essential for detecting differences in these and the contributions of the BBB (parameter γ) and relative sensitivity (*z*).

Because our Treatment Exposure and Sensitivity model is minimal and reduces mechanisms down to a few key parameters, it has great utility for fitting experimental data to estimate these parameters for individuals. Having these individual parameterizations is key to understanding the extent to which drug exposure and resistance each contributed to variations in outcome. In particular, quantifying drug sensitivity and exposure parameters for individual subjects within and between groups with additional therapies and PDX lines (better capturing interpatient, as well as intrapatient, heterogeneity) may be a promising avenue for future research. This will provide further insights for developing novel approaches to therapy optimization—including delivery—for individual patients.

## Data Availability Statement

All datasets generated for this study are included in the article/Supplementary Material.

## Ethics Statement

The animal study was reviewed and approved by Mayo Clinic Institutional Animal Care and Use Committee.

## Author Contributions

SM and JU contributed to the software and analysis. SM, JU, and BM contributed to the investigation. SM contributed to the preparation writing of the original draft. JS and KS contributed to the funding acquisition. All authors contributed to the conceptualization, methodology, and the writing—review and editing.

## Conflict of Interest

The authors declare that the research was conducted in the absence of any commercial or financial relationships that could be construed as a potential conflict of interest.
