# Supplementary material for: Quantifying Glioblastoma Drug Response Dynamics Incorporating Treatment Sensitivity and Blood Brain Barrier Penetrance From Experimental Data
Source: Front Physiol. 2020 Aug 21;11:830. doi: 10.3389/fphys.2020.00830 (PMC7472531; doi:10.3389/fphys.2020.00830)
Supplement: Supplementary file 1 [file Data_Sheet_1.PDF]

TESTING INITIAL PARAMETER GUESSES

In order to test the identifiability of parameters and the influence of our choice of initial guess for the fitting algorithm, we ran a series of fits using combinations of different initial guesses. Starting with the flank setting, we ran fits for parameters q, μ_H, and z, using low, medium, and high values as initial guesses. The initial proportion q of implanted PDX cells that has less sensitivity to the ADC ranges between 0 and 1 (as shown in Table 1 of the manuscript), but is generally assumed to be $<< 0.5$ (that is, a rare or small fraction). Thus in practice, it was bound between 10^{-10} and 10^{-2}, and for testing different initial guesses, we chose logarithmically distributed samples: low = 10^{-8} med = 10^{-5}, and high = 10^{-2}. The relative sensitivity z was bound between 0 and 1, and the range was sampled linearly: low = 0.2, med = 0.5, high = 0.8. Finally, the range of the ADC-mediated high sensitivity cell kill rate μ_H, which was bound between 0 and 10, was also sampled linearly: low = 2.5, med = 5, and high = 7.5. We ran the fitting algorithm using all combinations of these initial guess values; the resulting returned parameter values are shown in Table S1.

Table S1: **Fitted parameters for the treated Flank data using different initial guesses.** In the q, μ_H, z column, lmh = low medium high to show which combination of initial guesses was used. For example, mlh corresponds to the medium q, low μ_H, and high z initial guess values.

	Fit Results for log(q)					Fit Results for μH					Fit Results for z				
q, μH, z	Mouse 1	Mouse 2	Mouse 3	Mouse 4	Mouse 5	Mouse 1	Mouse 2	Mouse 3	Mouse 4	Mouse 5	Mouse 1	Mouse 2	Mouse 3	Mouse 4	Mouse 5
lll	-8.13	-2.00	-3.03	-4.46	-2.00	2.65	5.61	6.01	7.00	3.86	0.00	0.31	0.27	0.23	0.38
llm	-8.13	-2.00	-3.03	-4.46	-2.00	2.65	5.61	6.01	7.00	3.86	0.00	0.31	0.27	0.23	0.38
llh	-8.13	-2.00	-3.03	-4.46	-2.00	2.65	5.61	6.01	7.00	3.86	0.00	0.31	0.27	0.23	0.38
lml	-2.00	-2.00	-3.03	-4.46	-2.00	4.73	5.61	6.01	7.00	3.86	0.34	0.31	0.27	0.23	0.38
lmm	-8.13	-2.00	-3.03	-4.46	-2.00	2.65	5.61	6.01	7.00	3.86	0.00	0.31	0.27	0.23	0.38
lmh	-8.13	-2.00	-3.03	-4.46	-2.00	2.65	5.61	6.01	7.00	3.86	0.00	0.31	0.27	0.23	0.38
lhl	-2.00	-2.00	-3.03	-4.46	-2.00	4.73	5.61	6.01	7.00	3.86	0.34	0.31	0.27	0.23	0.38
lhm	-2.00	-2.00	-3.03	-4.46	-2.00	4.73	5.61	6.01	7.00	3.86	0.34	0.31	0.27	0.23	0.38
lhh	-8.13	-2.00	-3.03	-4.46	-2.00	2.65	5.61	6.01	7.00	3.86	0.00	0.31	0.27	0.23	0.38
mll	-8.13	-2.00	-3.03	-4.46	-2.00	2.65	5.61	6.01	7.00	3.86	0.00	0.31	0.27	0.23	0.38
mlm	-8.13	-2.00	-3.03	-4.46	-2.00	2.65	5.61	6.01	7.00	3.86	0.00	0.31	0.27	0.23	0.38
mlh	-8.13	-2.00	-3.03	-4.46	-2.00	2.65	5.61	6.01	7.00	3.86	0.00	0.31	0.27	0.23	0.38
mml	-2.00	-2.00	-3.03	-4.46	-2.00	4.73	5.61	6.01	7.00	3.86	0.34	0.31	0.27	0.23	0.38
mmm	-2.00	-2.00	-3.03	-4.46	-2.00	4.73	5.61	6.01	7.00	3.86	0.34	0.31	0.27	0.23	0.38
mmh	-2.00	-2.00	-3.03	-4.46	-2.00	4.73	5.61	6.01	7.00	3.86	0.34	0.31	0.27	0.23	0.38
mhl	-2.00	-2.00	-3.03	-4.46	-2.00	4.73	5.61	6.01	7.00	3.86	0.34	0.31	0.27	0.23	0.38
mhm	-2.00	-2.00	-3.03	-4.46	-2.00	4.73	5.61	6.01	7.00	3.86	0.34	0.31	0.27	0.23	0.38
mhh	-2.00	-2.00	-3.03	-4.46	-2.00	4.73	5.61	6.01	7.00	3.86	0.34	0.31	0.27	0.23	0.38
hll	-2.00	-2.00	-3.03	-4.46	-2.00	4.73	5.61	6.01	7.00	3.86	0.34	0.31	0.27	0.23	0.38
hlm	-2.00	-2.00	-3.03	-4.46	-2.00	4.73	5.61	6.01	7.00	3.86	0.34	0.31	0.27	0.23	0.38
hlh	-2.00	-2.00	-3.03	-4.46	-2.00	4.73	5.61	6.01	7.00	3.86	0.34	0.31	0.27	0.23	0.38
hml	-2.00	-2.00	-3.03	-4.46	-2.00	4.73	5.61	6.01	7.00	3.86	0.34	0.31	0.27	0.23	0.38
hmm	-2.00	-2.00	-3.03	-4.46	-2.00	4.73	5.61	6.01	7.00	3.86	0.34	0.31	0.27	0.23	0.38
hmh	-2.00	-2.00	-3.03	-4.46	-2.00	4.73	5.61	6.01	7.00	3.86	0.34	0.31	0.27	0.23	0.38
hhl	-2.00	-2.00	-3.03	-4.46	-2.00	4.73	5.61	6.01	7.00	3.86	0.34	0.31	0.27	0.23	0.38
hhm	-2.00	-2.00	-3.03	-4.46	-2.00	4.73	5.61	6.01	7.00	3.86	0.34	0.31	0.27	0.23	0.38
hhh	-2.00	-2.00	-3.03	-4.46	-2.00	4.73	5.61	6.01	7.00	3.86	0.34	0.31	0.27	0.23	0.38
min	-8.13	-2.00	-3.03	-4.46	-2.00	2.65	5.61	6.01	7.00	3.86	0.00	0.31	0.27	0.23	0.38
max	-2.00	-2.00	-3.03	-4.46	-2.00	4.73	5.61	6.01	7.00	3.86	0.34	0.31	0.27	0.23	0.38
mean	-4.04	-2.00	-3.03	-4.46	-2.00	4.04	5.61	6.01	7.00	3.86	0.22	0.31	0.27	0.23	0.38
median	-2.00	-2.00	-3.03	-4.46	-2.00	4.73	5.61	6.01	7.00	3.86	0.34	0.31	0.27	0.23	0.38

In all but one subject, we had good convergence—comparing the min and max fitted parameters shows that the only mouse that has a range > 0.005 is Mouse 1. In this mouse, it appears that the optimization converged to a different solution for a few initial value combinations. The plot of this alternative fit for the first mouse in the treated flank group (Figure S1) shows that it captures the ends well, including the second-to-last data point (which appears to be a bit of an outlier).

However, it does not fit the dynamics at the intermediate time points as well as the other, more commonly found fit for this mouse. Thus, the more frequently converged to solution appears to better approximate the data overall.

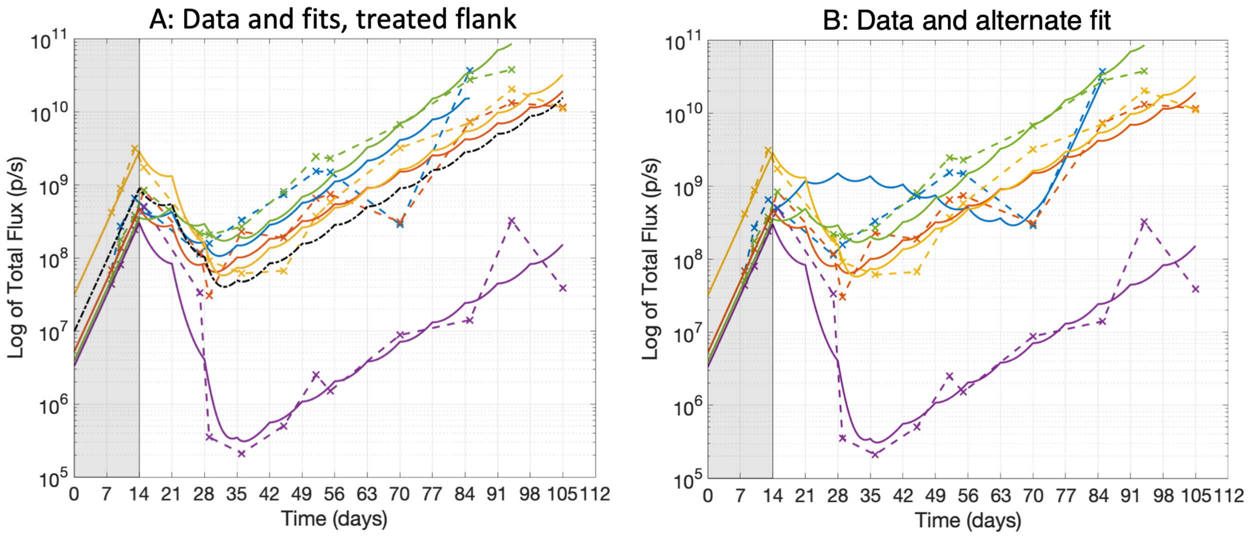

Figure S1: **A. Treated flank data and fits.** Solid blue line corresponds to the model simulation using the commonest parameter values fit for Mouse 1 ($q = 10^{-2}$, $\mu_H = 4.73$, and $z = 0.34$). **B. Alternative fit for Mouse 1.** Solid blue line corresponds to the model simulation using the alternate parameter values fit for Mouse 1 ($q = 10^{-8.13}$, $\mu_H = 2.65$, & $z = 0$). In both plots, blue x's connected by dashed line correspond to experimental data points.

We repeated this for the intracranial setting, with initial guesses for the three parameters fitted in that case: γ, q, and z. The same low, medium, and high values were used for q and z as in the flank setting. The proportion of tumor exposed to drug, γ, has a range from 0 to 1, and the initial guesses were sampled linearly: low = 0.2, med = 0.5, and high = 0.8. Again, the fitting algorithm was run with all combinations of initial guesses, and the resulting parameter fits are shown in Table S2, along with the maximum, minimum, mean, and median values for each subject.

In this case, we had greater variability in the fits for q and z, while γ was remarkably consistent. The variabilty is particularly noted in Mouse 3 and Mouse 5, where the range is wider and the mean and median differ more for the fits of q and z. The largest difference between the mean and median occured for Mouse 5, which died early in the set of experiments and thus had fewer data points to fit. This highlights the sensitivity in the choice of initial guess in the context of fewer data points in the time series when identifying parameters. Additionally, as the sensitivity analysis showed (in Section 3.1), the total tumor is relatively insensitive to parameters q and z early on and increases most over the first 50 days or 7 weeks (Figure 4A). Since the intracranial subjects all died before that, it may be that their influence on the model remained small enough to impact the pratical identifiability of parameters more precisely. Due to model sensitivity, we have made recommendations in the Discussion of the main manuscript regarding data collection to ensure parameters are practically identifiable using this approach.

Table S2: **Fitted parameters for the treated Intracranial data using different initial guesses.** In the q, μ_H, z column, lmh = low medium high to show which combination was used (e.g., mlh corresponds to the medium q, low μ_H, and high z initial guess values).

	Fit Results for log(q)					Fit Results for y					Fit Results for z				
q, y, z	Mouse 1	Mouse 2	Mouse 3	Mouse 4	Mouse 5	Mouse 1	Mouse 2	Mouse 3	Mouse 4	Mouse 5	Mouse 1	Mouse 2	Mouse 3	Mouse 4	Mouse 5
lll	-8.50	-10.00	-9.90	-9.07	-9.86	0.54	0.59	0.39	0.50	0.50	0.88	1.00	0.60	0.76	0.20
llm	-10.00	-10.00	-9.42	-9.57	-8.79	0.54	0.59	0.39	0.50	0.50	0.77	1.00	0.59	0.93	0.70
llh	-10.00	-8.59	-5.08	-9.89	-6.51	0.54	0.59	0.39	0.50	0.50	0.94	0.76	0.68	0.96	0.80
lml	-9.78	-9.44	-10.00	-9.88	-10.00	0.54	0.59	0.39	0.50	0.50	0.95	0.79	1.00	0.96	1.00
lmm	-9.70	-10.00	-9.99	-9.37	-10.00	0.54	0.59	0.39	0.50	0.50	0.98	1.00	1.00	0.91	1.00
lmh	-9.99	-9.39	-10.00	-8.01	-7.94	0.54	0.59	0.39	0.50	0.50	0.86	0.90	1.00	0.80	0.78
lhl	-10.00	-9.98	-10.00	-10.00	-9.98	0.54	0.59	0.39	0.50	0.50	1.00	1.00	1.00	1.00	1.00
lhm	-9.98	-10.00	-10.00	-9.98	-9.98	0.54	0.59	0.39	0.50	0.50	0.65	1.00	1.00	1.00	1.00
lhh	-9.93	-10.00	-8.35	-9.94	-2.65	0.54	0.59	0.39	0.50	0.50	1.00	1.00	1.00	1.00	1.00
mll	-10.00	-10.00	-4.90	-10.00	-9.95	0.54	0.59	0.39	0.50	0.50	1.00	1.00	0.71	1.00	0.99
mlm	-10.00	-9.80	-2.00	-7.62	-8.23	0.54	0.59	0.39	0.50	0.50	1.00	0.98	0.65	0.78	0.93
mlh	-9.44	-7.48	-2.00	-9.34	-9.99	0.54	0.59	0.39	0.50	0.50	0.93	0.92	0.65	0.96	1.00
mml	-9.49	-8.49	-9.32	-9.45	-9.46	0.54	0.59	0.39	0.50	0.50	0.94	0.78	1.00	0.92	1.00
mmm	-10.00	-8.11	-5.55	-9.56	-9.99	0.54	0.59	0.39	0.50	0.50	0.95	0.84	0.66	0.97	1.00
mmh	-8.66	-9.83	-2.00	-8.66	-9.98	0.54	0.59	0.39	0.50	0.50	0.97	1.00	0.65	0.97	1.00
mhl	-9.98	-9.45	-6.23	-9.18	-9.97	0.54	0.59	0.39	0.50	0.50	1.00	0.89	0.62	0.81	1.00
mhm	-8.45	-10.00	-5.36	-8.29	-9.95	0.54	0.59	0.39	0.50	0.50	1.00	1.00	0.64	0.82	1.00
mhh	-9.98	-7.66	-2.00	-7.55	-9.91	0.54	0.59	0.39	0.50	0.50	1.00	1.00	0.65	0.82	1.00
hll	-9.72	-9.57	-2.00	-9.59	-9.80	0.54	0.59	0.39	0.50	0.50	0.99	0.97	0.65	0.98	0.99
hlm	-9.80	-9.42	-2.00	-10.00	-5.47	0.54	0.59	0.39	0.50	0.50	0.99	0.97	0.65	0.98	0.63
hlh	-9.64	-9.16	-2.00	-8.22	-9.73	0.54	0.59	0.39	0.50	0.50	0.99	0.76	0.65	0.92	0.99
hml	-9.69	-9.49	-2.00	-9.44	-7.05	0.54	0.59	0.39	0.50	0.50	0.98	0.96	0.65	0.73	0.64
hmm	-10.00	-10.00	-2.00	-6.55	-6.15	0.54	0.59	0.39	0.50	0.50	1.00	1.00	0.65	0.81	0.76
hmh	-7.65	-9.05	-2.00	-6.46	-6.36	0.54	0.59	0.39	0.50	0.50	1.00	0.99	0.65	0.92	0.91
hhl	-10.00	-8.17	-2.00	-7.91	-6.20	0.54	0.59	0.39	0.50	0.50	1.00	0.85	0.65	0.86	0.64
hhm	-7.13	-7.86	-2.00	-6.69	-4.76	0.54	0.59	0.39	0.50	0.50	0.92	0.87	0.65	0.80	0.98
hhh	-7.01	-9.99	-2.00	-6.83	-6.94	0.54	0.59	0.39	0.50	0.50	0.95	1.00	0.65	0.88	0.86
min	-10.00	-10.00	-10.00	-10.00	-10.00	0.54	0.59	0.39	0.50	0.50	0.65	0.76	0.59	0.73	0.20
max	-7.01	-7.48	-2.00	-6.46	-2.65	0.54	0.59	0.39	0.50	0.50	1.00	1.00	1.00	1.00	1.00
mean	-9.43	-9.29	-5.19	-8.78	-8.35	0.54	0.59	0.39	0.50	0.50	0.95	0.93	0.74	0.90	0.88
median	-9.80	-9.49	-4.90	-9.34	-9.73	0.54	0.59	0.39	0.50	0.50	0.98	0.98	0.65	0.92	0.99
