# Supplementary material for: Quantifying Glioblastoma Drug Response Dynamics Incorporating Treatment Sensitivity and Blood Brain Barrier Penetrance From Experimental Data
Source: Front Physiol. 2020 Aug 21;11:830. doi: 10.3389/fphys.2020.00830 (PMC7472531; doi:10.3389/fphys.2020.00830)
Supplement: Supplementary file 2 [file Data_Sheet_2.pdf]

A Analytical Solution to Model

Here we present the derivation of the analytical solution for model system (??). Recall that the system consists of the following differential equations, which describe the growth of two tumor cell populations with differential drug sensitivity, H and L, and drug level, A:

$$\frac{dH}{dt} = \rho H - \gamma \mu_H A H \tag{1a}$$

$$\frac{dL}{dt} = \rho L - \gamma \mu_L A L \tag{1b}$$

$$\frac{dA}{dt} = \sum_{n=1}^{N} \hat{A}(n) \delta\left(t - 7n\right) - \lambda A \tag{1c}$$

where $\hat{A}$ is a vector containing the consecutive drug doses administered, such that $\hat{A}(n)$ is the dose on the nth pulse (of which there are at most N drug pulses), and $7n$ denotes the time of the nth pulse (every 7 days). The parameters are given in Table **??** of the main text.

Because the equation for A in the system (1) is independent of H and L, we first solve (1c) for A. First, observe the following rearrangement:

$$\frac{dA}{dt} = \sum_{n=1}^{N} \hat{A}(n) \delta\left(t - 7n\right) - \lambda A \tag{2}$$

$$\implies A' + \lambda A = \sum_{n=1}^{N} \hat{A}(n) \delta\left(t - 7n\right) \tag{3}$$

Notice that we can use the method of integrating factors, introducing a factor of $e^{\lambda t}$:

$$e^{\lambda t} A' + \lambda e^{\lambda t} A = e^{\lambda t} \sum_{n=1}^{N} \hat{A}(n) \delta\left(t - 7n\right) \tag{4}$$

so that

$$\int \left(e^{\lambda t} A' + \lambda e^{\lambda t} dt A\right) = \int e^{\lambda t} \sum_{n=1}^{N} \hat{A}(n) \delta\left(t - 7n\right) dt \tag{5}$$

$$= \int \left(e^{\lambda t} A\right)' = \int e^{\lambda t} \sum_{n=1}^{N} \hat{A}(n) \delta\left(t - 7n\right) dt \tag{6}$$

Integrating the left hand side, and moving the integral inside the sum on the right hand side:

$$e^{\lambda t} A = \sum_{n=1}^{N} \hat{A}(n) \int e^{\lambda t} \delta\left(t - 7n\right) dt \tag{7}$$

Then, integrating the right hand side, we have

$$e^{\lambda t} A = \sum_{n=1}^{N} \hat{A}(n) e^{7n\lambda} \theta\left(t - 7n\right), \tag{8}$$

where θ is the heaviside function.

Now, since $\lambda = \ln(2)/7$ (i.e., the half life is 7 days), we have the coefficient

$$e^{7n\lambda} = e^{7n\ln(2)/7} = e^{\ln(2)n} = 2^n, \tag{9}$$

so the right hand side of (8) becomes

$$e^{\lambda t}A = \sum_{n=1}^{N} 2^n \hat{A}(n)\theta\left(t - 7n\right). \tag{10}$$

Rearranging, we have our solution for A:

$$A = \sum_{n=1}^{N} 2^n \hat{A}(n)e^{-\lambda t}\theta\left(t - 7n\right). \tag{11}$$

Now we solve for H and L, and use the fact that (1a) and (1b) differ only by parameter μ_H versus μ_L to obtain both simultaneously:

$$\frac{dH}{dt} = \rho H - \gamma\mu_H AH \tag{12}$$

$$\implies \frac{dH}{H} = \rho dt - \gamma\mu_H A(t)dt \tag{13}$$

Integrating both sides:

$$\int \frac{dH}{H} = \rho \int dt - \gamma\mu_H \int A(t)dt \tag{14}$$

$$\ln(H) = \rho t - \gamma\mu_H \int A(t)dt + c_1, \tag{15}$$

where c_1 is a constant of integration to be determined from the initial condition.

Since the solution for H in (15) depends on $\int A(t)dt$, we must integrate (11):

$$\int A(t)dt = \int \sum_{n=1}^{N} 2^n \hat{A}(n)e^{-\lambda t}\theta\left(t - 7n\right)dt \tag{16}$$

$$= \sum_{n=1}^{N} 2^n \hat{A}(n) \int e^{-\lambda t}\theta\left(t - 7n\right)dt \tag{17}$$

To use integration by parts, such that $\int u'v = uv - \int uv'$, choose

$$u = e^{-\lambda t}, \qquad v = \theta(t - 7n) \tag{18}$$

$$u' = -\lambda e^{-\lambda t}dt, \qquad v' = \delta(t - 7n)dt \tag{19}$$

Then we have:

$$\int e^{-\lambda t}\theta\left(t-7n\right)dt = -\frac{1}{\lambda}\int -\lambda e^{-\lambda t}\theta\left(t-7n\right)dt \tag{20}$$

$$= -\frac{1}{\lambda}\left(e^{-\lambda t}\theta\left(t-7n\right) - \int e^{-\lambda t}\delta\left(t-7n\right)dt\right) \tag{21}$$

$$= -\frac{1}{\lambda}\left(e^{-\lambda t}\theta\left(t-7n\right) - e^{-7n\lambda}\theta\left(t-7n\right)\right) + c_n \tag{22}$$

$$= \frac{-\left(e^{-\lambda t} - e^{7n\lambda}\right)\theta\left(t-7n\right)}{\lambda} + c_n \tag{23}$$

$$= \frac{\left(e^{7n\lambda} - e^{-\lambda t}\right)\theta\left(t-7n\right)}{\lambda} + c_n \tag{24}$$

where c_n is a constant of integration. Thus, (17) becomes:

$$\int A(t)dt = \sum_{n=1}^{N} 2^n \hat{A}(n)\frac{\left(e^{7n\lambda} - e^{-\lambda t}\right)\theta\left(t-7n\right)}{\lambda} + c_2, \tag{25}$$

where c_2 is the sum of all the c_n.

Now we can insert (25) into (15), giving

$$\ln(H) = \rho t - \gamma\mu_H \sum_{n=1}^{N} 2^n \hat{A}(n)\frac{\left(e^{7n\lambda} - e^{-\lambda t}\right)\theta\left(t-7n\right)}{\lambda} + c_2 + c_1 \tag{26}$$

$$\text{or, letting } c_0 = c_2 + c_1 \tag{27}$$

$$\ln(H) = \rho t - \gamma\mu_H \sum_{n=1}^{N} 2^n \hat{A}(n)\frac{\left(e^{7n\lambda} - e^{-\lambda t}\right)\theta\left(t-7n\right)}{\lambda} + c_0 \tag{28}$$

Solving for H then, we find

$$e^{\ln(H)} = \exp\left(\rho t - \gamma\mu_H \sum_{n=1}^{N} 2^n \hat{A}(n)\frac{\left(e^{7n\lambda} - e^{-\lambda t}\right)\theta\left(t-7n\right)}{\lambda} + c_0\right) \tag{29}$$

$$H = e^{c_0}\exp\left(\rho t - \gamma\mu_H \sum_{n=1}^{N} 2^n \hat{D}(n)\frac{\left(e^{7n\lambda} - e^{-\lambda t}\right)\theta\left(t-7n\right)}{\lambda}\right) \tag{30}$$

Denoting $\exp(c_0) = H_0$, we arrive at our solution for H:

$$H = H_0\exp\left(\rho t - \gamma\mu_H \sum_{n=1}^{N} 2^n \hat{A}(n)\frac{\left(e^{7n\lambda} - e^{-\lambda t}\right)\theta\left(t-7n\right)}{\lambda}\right). \tag{31}$$

The solution for L is similar:

$$L = L_0\exp\left(\rho t - \gamma\mu_L \sum_{n=1}^{N} 2^n \hat{A}(n)\frac{\left(e^{7n\lambda} - e^{-\lambda t}\right)\theta\left(t-7n\right)}{\lambda}\right). \tag{32}$$

Now, we assume that the H cells are more sensitive to drug than the L cells, such that $\mu_H > \mu_L$. Defining z to be the ratio $z = \mu_L/\mu_H$, we can substitute $\mu_L = z\mu_H$, and in our fitting process, solve for this degree of differential sensitivity between the H and L cell populations.

Additionally, for fitting purposes, we can combine the cells into one total population: $C = H + L$. To do this, we write the initial condition $C_0 = H_0 + L_0$, and let $H_0 = qC_0$ such that q represents the proportion of C_0 that is made up of cells that are more drug–sensitive. This gives:

$$\begin{aligned} C = qC_0 \exp\left(\rho t - \gamma\mu_H \sum_{n=1}^{N} 2^n \hat{A}(n) \frac{\left(e^{7n\lambda} - e^{-\lambda t}\right)\theta\left(t - 7n\right)}{\lambda}\right) \\ + (1-q)C_0 \exp\left(\rho t - \gamma z\mu_H \sum_{n=1}^{N} 2^n \hat{A}(n) \frac{\left(e^{7n\lambda} - e^{-\lambda t}\right)\theta\left(t - 7n\right)}{\lambda}\right) \end{aligned} \quad (33)$$

Because this is a lengthy expression, in the main text we write

$$C(t) = C_0 e^{\rho t}\left(q e^{-\gamma\mu_H \int A(t)dt} + (1-q)e^{-\gamma z\mu_H \int A(t)dt}\right), \quad (34)$$

where $\int Adt$ denotes the summation in (17).
